# Retinal Blood Flow Velocity Change in Parafoveal Capillary after Topical Tafluprost Treatment in Eyes with Primary Open-angle Glaucoma

**DOI:** 10.1038/s41598-017-05258-4

**Published:** 2017-07-10

**Authors:** Yuto Iida, Tadamichi Akagi, Hideo Nakanishi, Hanako Ohashi Ikeda, Satoshi Morooka, Kenji Suda, Tomoko Hasegawa, Satoshi Yokota, Munemitsu Yoshikawa, Akihito Uji, Nagahisa Yoshimura

**Affiliations:** Department of Ophthalmology and Visual Sciences, Kyoto University Graduate School of Medicine, Kyoto, Japan

## Abstract

Although ocular circulation at the retina and optic disc is known to be associated with the pathology of glaucoma, direct measurement of blood flow velocity has been difficult to obtain. This prospective observational study enrolled 11 consecutive patients with treatment-naïve primary open-angle glaucoma (POAG) and 11 healthy subjects, and the effects of topical tafluprost treatment on ocular circulation were examined at baseline and at 1, 4, and 12 weeks after initiating treatment with topical tafluprost on POAG patients using multiple modalities, which include adaptive optics scanning laser ophthalmoscopy (AOSLO). Baseline mean intraocular pressure (IOP) was significantly higher and mean parafoveal blood flow velocity (pBFV) was significantly lower in POAG eyes than in healthy eyes. Mean IOP was significantly decreased (1 week, −19.1%; 4 weeks, −17.7%; and 12 weeks, −23.5%; all *P* < 0.001) and mean pBFV was significantly increased from the baseline at all follow-up periods after initiating treatment (1 week, 14.9%, *P* = 0.007; 4 weeks, 21.3%, *P* < 0.001; and 12 weeks, 14.3%, *P* = 0.002). These results reveal that tafluprost may not only lower IOP but may also improve retinal circulation in POAG eyes and AOSLO may be useful to evaluate retinal circulatory change after treatment.

## Introduction

Intraocular pressure (IOP) is considered to be the most important risk factor for the development and progression of glaucoma^1, 2^. On the other hand, ocular blood flow might also be related to glaucoma. Although a number of previous studies have shown that ocular circulation of the retina and optic disc is associated with glaucoma^3–10^, the effect of IOP reduction on ocular blood flow in glaucomatous eyes is controversial^11–16^.

Adaptive optics scanning laser ophthalmoscopy (AOSLO) is a promising technology that allows for the non-invasive monitoring of leukocyte movements and the direct measurement of the parafoveal retinal blood flow velocity (pBFV) without the use of contrast dyes^17, 18^. Retinal vessel diameter (RVD) may be associated with glaucoma pathogenesis and can be measured using spectral-domain optical coherence tomography (OCT) with high reproducibility^19–22^. Laser speckle flowgraphy (LSFG) can measure relative optic disc blood flow using the laser speckle phenomenon and its measurement, mean blur rate (MBR), has been used for optic disc blood flow evaluation^6, 23–27^. However, it is unknown if parafoveal retinal blood flow velocity (pBFV) in glaucoma patients is different from that in healthy subjects and if it is affected by glaucoma treatment, or if it changes equally as the other parameters.

**Figure 1:**
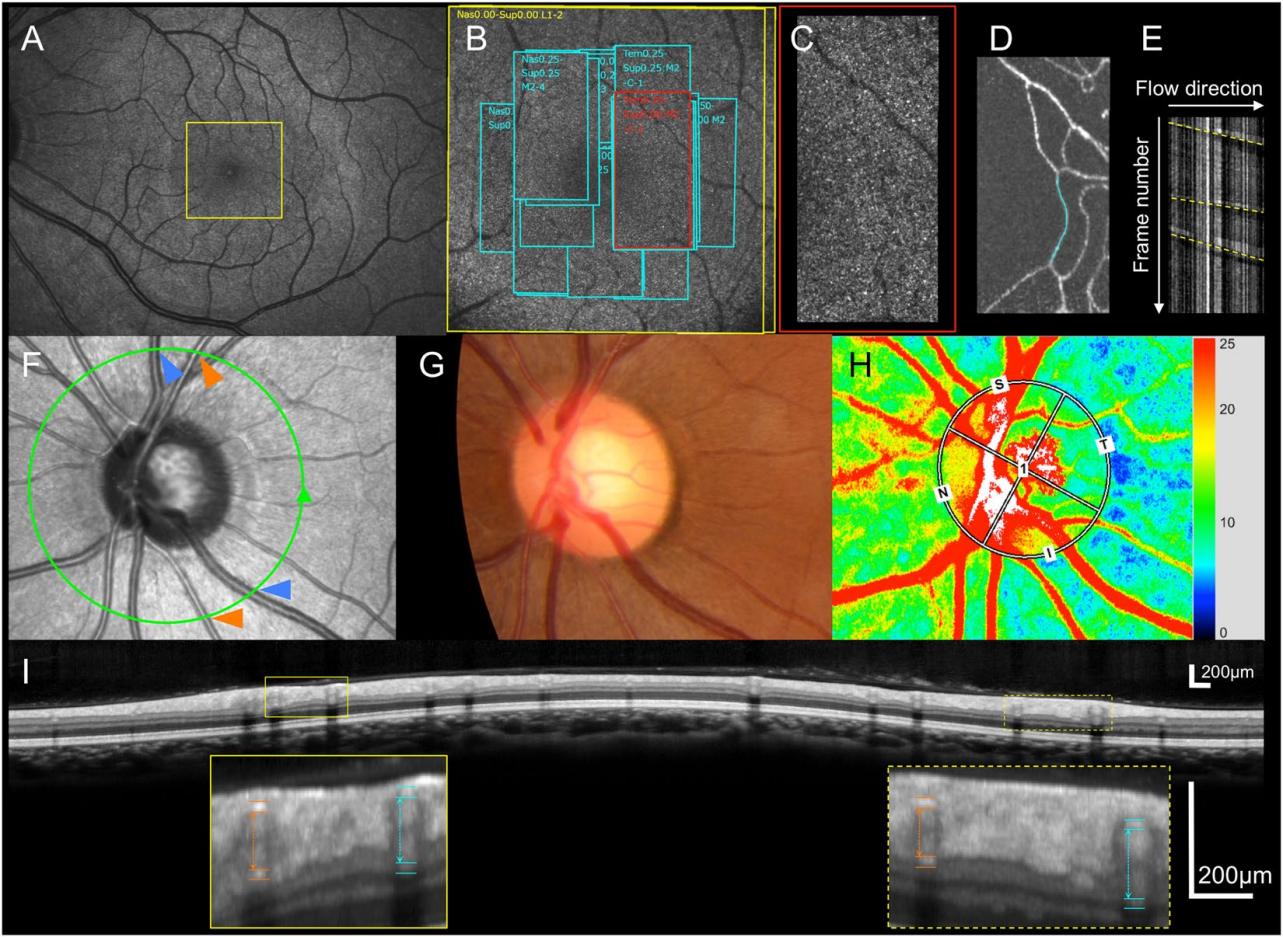
An example of three types of measurements in a normal subject. (**A**) Wide field scanning laser ophthalmoscopy (SLO) image. The detailed scanning area is indicated with a yellow outlined rectangle. (**B**) Adaptive optics SLO (AOSLO) image as shown by the yellow outlined rectangle in **A**. Rectangular areas outlined in aqua and red indicate locations of AOSLO video recording. (**C**) An AOSLO image as shown by the red outlined rectangle in (**B**). (**D**) A montage image obtained from AOSLO video using motion contrast enhancement technique. The target vessel is shown in aqua. (**E**) A spatiotemporal image of the target vessel shown as aqua in (**D**). The longitudinal axis represents frame number of consecutive images, and the horizontal axis represents vessel length and flow direction. White bands represent leukocytes and following dark bands represent aggregated erythrocytes in the capillary vessel. Dotted yellow lines indicate anterior ends of aggregated erythrocytes. Reciprocals of the slope of the lines represent parafoveal blood flow velocity (pBFV). (**F**) An SLO image of the optic disc. (**G**) Fundus photograph around the optic disc. (**H**) Laser speckle flowgraphy image around the optic disc. **I**, Circumpapillary scan of spectral-domain optical coherence tomography shown as the green circle in (**F**). The top and the bottom of vessel walls are depicted as hyper-reflectivities. Arterial and venous inner diameters are shown as orange and aqua dotted double-headed arrows, respectively.

Some reports have shown that tafluprost, a prostaglandin (PG) F_2F_ derivative, may increase ocular circulation^27, 28^. However, many questions regarding its effects on ocular circulation remain to be answered, such as whether the retinal blood flow of the glaucomatous eyes could be affected by topical tafluprost treatment, whether the optic disc blood flow in eyes with other than myopic disc type could change in response to tafluprost treatment, whether the circulations of the retina and optic disc change simultaneously, or which kinds of measurements are likely to detect the changes in ocular circulation. In this prospective study, the effects of topical tafluprost treatment on ocular circulation were investigated using three different types of measurements in treatment-naïve primary open-angle glaucoma (POAG) patients: parafoveal retinal blood flow using AOSLO, RVD using OCT, and optic disc blood flow using LSFG (Fig. 1).

## Results

### Subjects

Fifteen eyes with POAG were enrolled in the present study, but four eyes were excluded because of insufficient AOSLO image quality. Eleven eyes of 11 POAG patients and 11 eyes of 11 healthy subjects were included in this study (Table 1). We could not obtain the data from one patient at 12 weeks after initiating tafluprost treatment because of one patient’s refusal to continue participation. We also could not use AOSLO images at 12 weeks of follow-up period of one patient because of poor image quality. We excluded these data from the analysis. The detailed protocol and raw data of the study are available at http://www.nature.com/srep.

**Table 1:** Baseline Characteristics of Patients with Primary Open Angle Glaucoma and Healthy Subjects.

	Healthy subjects	POAG patients	*P* value*
Number (eyes)	11	11	
Sex (male)	6	7	0.67
Age (years)	50.3 ± 11.8	54.9 ± 8.4	0.30
Axial length (mm)	23.98 ± 0.72	25.33 ± 1.48	0.056
BCVA (LogMAR)	−0.18 ± 0.07	−0.16 ± 0.04	0.41
VF mean deviation (dB)	N/A	−5.23 ± 4.41	N/A
Systolic blood pressure (mmHg)	121.3 ± 10.6	116.0 ± 18.0	0.60
Diastolic blood pressure (mmHg)	72.3 ± 6.8	69.2 ± 11.0	0.61
Intraocular pressure (mmHg)	13.3 ± 1.2	17.1 ± 2.3	0.002
Ocular perfusion pressure (mmHg)	45.3 ± 4.4	39.5 ± 8.3	0.21
pBFV (mm/sec)	1.35 ± 0.33	1.05 ± 0.16	0.014
MBR_T_ (AU)	14.5 ± 3.2	11.8 ± 2.7	0.047
RVD_A_ (μm)	109.3 ± 7.4	98.0 ± 12.4	0.019
RVD_V_ (μm)	149.9 ± 12.6	131.9 ± 11.7	0.003

Values comprise mean ± standard deviation.

POAG = primary open-angle glaucoma; BCVA = best corrected visual acuity; LogMAR = logarithm of minimum angle of resolution; VF = visual field; pBFV = parafoveal retinal blood flow velocity; MBR_T_ = mean blur rate in the tissue area of the optic nerve head; RVD_A_ = retinal vessel diameter of artery; RVD_V_ = retinal vessel diameter of vein; N/A = not applicable.

*Comparison was performed using the chi-square test for sex and the unpaired t-test for the other parameters.

### Baseline Evaluation of Eyes with POAG and Comparison with Healthy Subjects

The baseline characteristics of the POAG patients and the healthy subjects are shown in Table 1. The mean pBFV, MBR in the tissue area of the optic disc (MBR_T_), and RVD were significantly lower in POAG eyes than in healthy eyes (mean pBFV, 1.05 ± 0.16 mm/sec versus 1.35 ± 0.33 mm/sec, *P* = 0.014; MBR_T_, 11.8 ± 2.7 AU versus 14.5 ± 3.2 AU, *P* = 0.047; RVD_A_, 98.0 ± 12.4 μm versus 109.3 ± 7.4 μm, *P* = 0.019; and RVD_V_, 131.9 ± 11.7 μm versus 149.9 ± 12.6 μm, *P* = 0.003). The mean baseline IOP was significantly higher in POAG eyes than in healthy subjects (17.1 ± 2.3 mmHg versus 13.3 ± 1.2 mmHg, *P* = 0.002), whereas no significant differences between the two groups in sBP (116.0 ± 18.0 mmHg versus 121.3 ± 10.6 mmHg, *P* = 0.60); dBP (69.2 ± 11.0 mmHg versus 72.3 ± 6.8 mmHg, *P* = 0.61); and OPP (39.5 ± 8.3 mmHg versus 45.3 ± 4.4 mmHg, *P* = 0.21) were observed. There were no significant differences between age, sex, axial length, and BCVA of the two groups.

### Longitudinal Changes after Topical Tafluprost Initiation

**Figure 2:**
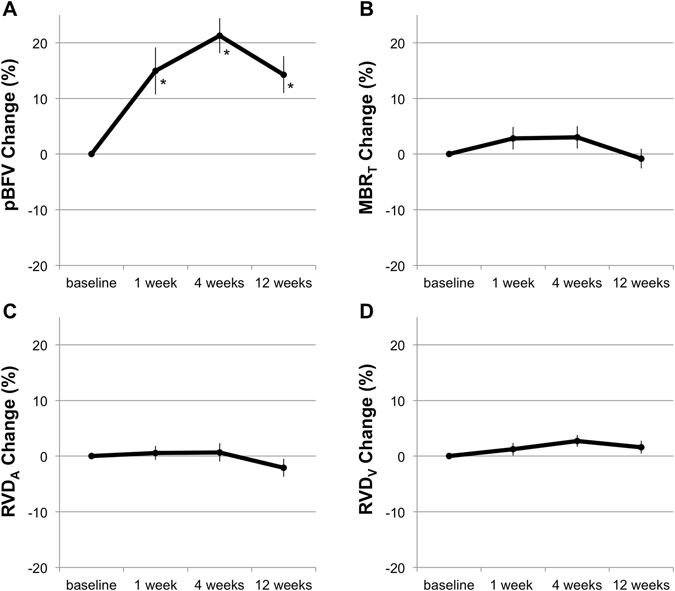
Percentage changes of circulatory parameters. Parafoveal blood flow velocity (pBFV, **A**), mean blur rate in the tissue area of the optic disc (MBR_T_, **B**), and retinal vessel diameters of artery (RVD_A_, **C**) and vein (RVD_V_, **D**) in eyes with primary open-angle glaucoma when compared with the baseline. Only the pBFV (**A**) significantly increased after topical tafluprost induction. *P shows statistical significance using a paired t-test adjusted by Bonferroni’s correction in measured values.

The longitudinal changes of pBFV, LSFG MBR_T_, RVD, IOP, BP, and OPP of the 11 POAG eyes are shown in Table 2 and Fig. 2. Identical vessels could be depicted in AOSLO images at all follow-up examinations. The mean IOP after topical tafluprost induction was significantly decreased at all follow-up periods from the baseline IOP (1, 4, and 12 weeks, *P* < 0.001), whereas there were no significant changes between the baseline and each follow-up period in sBP, dBP, and OPP (*P* = 0.071, *P* = 0.75, and *P* = 0.68). The mean pBFV after topical tafluprost induction was significantly increased at all follow-up periods from the baseline (*P* = 0.007, *P* < 0.001, and *P* = 0.002). There were no significant changes between the baseline and each follow-up period in MBR_T_, RVD_A_, and RVD_V_ (*P* = 0.14, *P* = 0.37, and *P* = 0.22).

**Table 2:** Change in each parameter before and after tafluprost initiation.

	baseline	1 week	4 weeks	12 weeks	post hoc analysis, comparison with baseline*			
					repeated measures ANOVA	1 week	4 weeks	12 weeks
pBFV (mm/sec)	1.05 ± 0.16	1.21 ± 0.26	1.28 ± 0.24	1.19 ± 0.21	*P* < 0.001	*P* = 0.007^†^	*P* < 0.001^†^	*P* = 0.002^†^
MBR_T_ (AU)	11.8 ± 2.7	12.2 ± 3.1	12.3 ± 3.3	11.9 ± 3.2	*P* = 0.14			
RVD_A_ (μm)	98.0 ± 12.4	98.5 ± 13.1	98.6 ± 13.5	93.7 ± 11.6	*P* = 0.37			
RVD_V_ (μm)	131.9 ± 11.7	133.6 ± 13.7	135.7 ± 15.3	134.8 ± 16.7	*P* = 0.22			
IOP (mmHg)	17.1 ± 2.3	13.7 ± 1.5	14.0 ± 2.0	14.4 ± 2.0	*P* < 0.001	*P* < 0.001^†^	*P* < 0.001^†^	*P* < 0.001^†^
OPP (mmHg)	39.5 ± 8.3	40.8 ± 9.3	42.9 ± 8.6	44.1 ± 9.4	*P* = 0.68			
sBP (mmHg)	116.0 ± 18.0	111.0 ± 19.5	118.4 ± 20.2	120.9 ± 18.9	*P* = 0.071			
dBP (mmHg)	69.2 ± 11.0	67.2 ± 12.1	68.8 ± 15.9	71.1 ± 13.0	*P* = 0.75			

Values comprise mean ± standard deviation.

ANOVA = analysis of variance; pBFV = parafoveal retinal blood flow velocity; MBR_T_ = mean blur rate in the tissue area of the optic nerve head; RVD_A_ = retinal vessel diameter of artery; RVD_V_ = retinal vessel diameter of vein; IOP = intraocular pressure; OPP = ocular perfusion pressure; sBP = systolic blood pressure; dBP = diastolic blood pressure.

^*^Comparison was performed using the paired t-test.

^†^Significant differences after Bonferroni’s correction are indicated.

**Figure 3:**
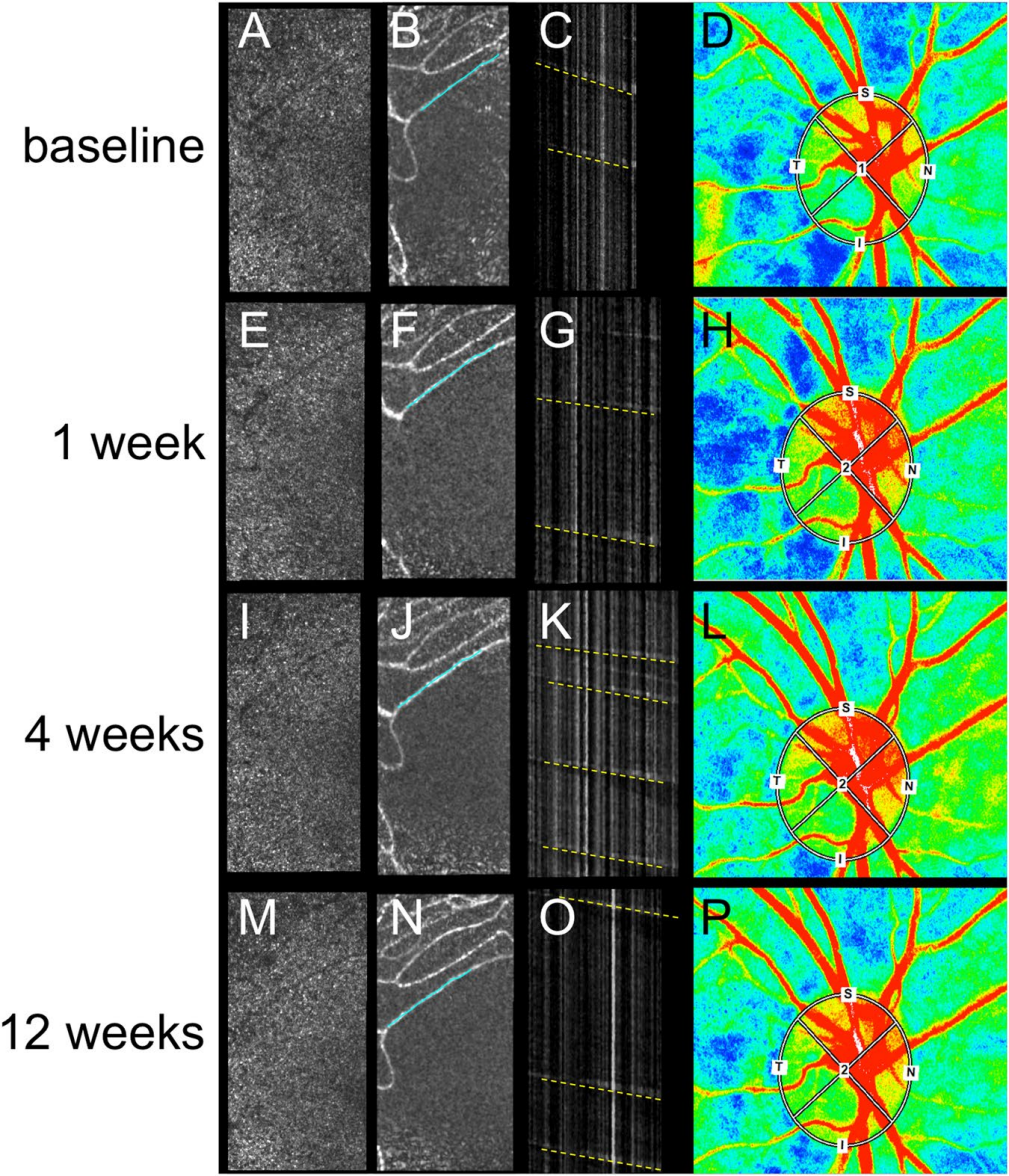
Representative case with primary open-angle glaucoma before and after tafluprost induction. (**A,E,I** and **M**) Adaptive optics scanning laser ophthalmoscopy (AOSLO) images. (**B,F,J and N**) Montage images from AOSLO videos. The identical vessels are depicted in all follow-up examinations (aqua lines). (**C,G,K and O**) Spatiotemporal images of the target vessel. Parafoveal blood flow velocity (pBFV), the reciprocals of the slopes of yellow dotted lines, increased in comparison to the baseline measurement (baseline, 1.11 mm/s; 1 week, 1.33 mm/s; 4 weeks, 1.36 mm/s; and 12 weeks, 1.23 mm/s). (**D,H,L** and **P**) Mean blur rate in the tissue area of the optic disc (MBR_T_) measurements using laser speckle flowgraphy (LSFG; baseline, 14.9 AU; 1 week, 16.1 AU; 4 weeks, 15.4 AU; and 12 weeks, 15.1 AU).

The longitudinal images of pBFV and MBR_T_ from a representative case are shown in Fig. 3. The raw videos at the baseline and 4 weeks after tafluprost induction are shown in Supplementary Video S1. The reciprocal of the slope in the ST images expressed pBFV (mm/sec) in the target vessel. It could be confirmed that the pBFV increased after starting tafluprost when compared to the baseline measurement.

## Discussion

In the current study, three different parameters associated with ocular blood flow were investigated in treatment-naïve eyes with POAG to clarify differences and similarities in response to tafluprost treatment among different types of established parameters. The pBFV measured by AOSLO, the MBR_T_ measured by LSFG, and RVD_A_ and RVD_V_ measured by OCT were significantly lower in untreated eyes with POAG than in healthy eyes. The longitudinal pBFV changes were successfully measured using the AOSLO system; furthermore, we confirmed that the pBFV increased maximally up to 21.9% 3 months after topical tafluprost treatment in the treatment-naïve POAG patients, while changes in the RVD and LSFG MBR_T_ were not significant in the same eyes.

Although ocular circulations in glaucomatous eyes have been intensively investigated using various methods in previous studies so far, AOSLO in the current study is fundamentally different from other techniques. AOSLO enables direct identification of moving leukocytes and aggregated erythrocytes in a targeted vessel, which permits us to measure absolute values of retinal vessel velocity with a unit of mm/sec repeatedly in the same vessel non-invasively with high reproducibility^17, 18, 29^. Significant increase in pBFV was detected after tafluprost treatment at all follow-up periods, which suggests that AOSLO may have the capability to detect a minute change in blood flow, and that topical tafluprost may be beneficial in improving the ocular circulation in POAG patients. Although the IOP was significantly reduced, the increase in OPP was not significant in our study. This suggests that an increase in drug efficacy as well as OPP may affect the parafoveal capillary blood flow, although it is unknown whether this blood flow increase was derived from the increase in OPP, drug efficacy, or both. For a more precise discussion, direct monitoring of blood pressure at the eye level may be needed because the OPP we used in this study was merely an estimate value from an empirically used method^30^. On the other hand, it should be noted that pBFV does not measure blood flow volume, but measures velocity. Because the AOSLO resolution was insufficient to accurately measure parafoveal capillary luminal diameters, blood flow volume could not be determined. Further high-resolution modalities might resolve these questions in the future.

Ocular blood flow autoregulation is known to correspond to changes in OPP and keep blood flow relatively constant, and some studies suggest that it is disrupted in glaucomatous eyes^31, 32^. Retina and optic disc blood flow, in which endothelin-1 plays an important role, is mainly involved in the regulation of local mechanisms^33^. Ishii *et al*.^34^ reported that topical latanoprost increased optic disc blood flow and its effect was independent of its IOP-reducing effect. Tafluprost topical treatment significantly increased retinal blood flow measured using a laser Doppler velocimetry system in cats^28^. Tsuda *et al*.^27^ showed that topical tafluprost significantly increased MBR in myopic optic discs in glaucomatous eyes. The vasodilator effects of tafluprost on endothelin-1-induced vasoconstriction was more powerful than those of latanoprost and travoprost in rabbits^35^, which is why we used tafluprost in our study. The LSFG measurements did not significantly change after tafluprost initiation in the current study, which is inconsistent with the report by Tsuda *et al*.^27^ This discrepancy can probably be explained by the differences in the included eyes and the timing of examination; their study only included eyes with a myopic optic disc type and only investigated the response at relatively short periods of time for up to 120 minutes. Thus, measurements associated with ocular blood flow are not always comparable even in similar conditions. Alternatively, AOSLO may be a better method for detecting minute changes in blood flow because it directly measures blood velocity.

We also found a discrepancy between pBFV and MBR_T_ in response to tafluprost treatment, but the reason for this discrepancy could not be clarified in the current study. It has been suggested that autoregulation might be different in the ONH and in the retina^10, 36^. One possible reason for the discrepancy is that the effect of tafluprost on ocular circulation might be different depending on the location, parafoveal retinal capillary vessel or capillary vessel in the optic disc. Another possibility depends on the different types of evaluation methods used for ocular blood flow. The blood flow velocity was directly measured in a specific capillary vessel in pBFV measurement, whereas only relative values of blood flow could be measured in a specific area, not the vessel itself, in MBR_T_ measurement. Further studies are needed to clarify the importance of this issue.

Whether an increase in pBFV affects current visual function or subsequent functional deterioration remains unknown. Krupin *et al*.^37^ showed that treatment with topical alpha 2-adrenergic agonist brimonidine (0.2%) reduced visual field progression rate compared to β-adrenoceptor blocker timolol (0.5%) in low-pressure glaucoma, which suggests that it may have a neuroprotective effect. Feke *et al*.^38^ reported that topical brimonidine treatment significantly improved impaired retinal vascular autoregulation in eyes with normal tension glaucoma, suggesting that the normalization of retinal vascular autoregulation is related to a neuroprotective effect. In the current study, differences in visual acuity before and after tafluprost treatment were not seen, and visual field testing was not performed after treatment. Further study is needed to clarify whether improvement of parafoveal capillary vessel velocity affects visual function.

In our study, the RVD did not show significant changes after initiating tafluprost treatment. Kurvinen *et al*.^39^ showed that IOP reduction after glaucoma surgery resulted in the reduction of retinal arterial diameter in exfoliation glaucoma patients using the retinal vessel analyzer, which made use of a fundus camera. It should be noted that length measurements on the coronal plane were affected by axial lengths and that IOP reduction could induce reduction in axial length, which might lead to overestimation of vessel width^40^. Recently, measurement of vessel diameters using OCT has been an attractive approach to overcome some of the limitations of classical fundus imaging^22, 41^. Our small sample size showed, at least, no obvious change after initiating tafluprost treatment using OCT-based RVD measurements.

This study has some limitations in addition to the relatively small sample size. We examined the effect of only one drug, tafluprost, because of its possible effects on blood flow improvements which has been demonstrated in previous studies. Second, we examined the effects of tafluprost only in POAG patients and not in healthy subjects. Thus, we could not determine whether the pBFV increase after tafluprost treatment is a unique phenomenon in POAG patients with disruptions in autoregulation or whether it can be applied to normal subjects as well. Third, we examined pBFV only in one selected vessel in each eye, not in all parafoveal capillaries. Further studies are needed to address these limitations.

In conclusion, by using AOSLO, we evaluated parafoveal retinal blood flow directly and non-invasively in eyes with POAG. Parafoveal retinal blood flow, optic disc blood flow, and RVD were significantly lower in POAG eyes than in normal subjects, and only the parafoveal retinal blood flow showed significant long-term increase after tafluprost initiation. Tafluprost may be effective not only in lowering IOP, but also in increasing retinal blood flow. Furthermore, direct measurement of vessel velocity using AOSLO may be a powerful method for evaluation of ocular circulation, not only in patients with metabolic disorders, but also in glaucoma patients.

## Methods

### Study Design and Patients

We performed a prospective observational study from October 21, 2014 to December 31, 2015 at the Glaucoma Clinic of Kyoto University Hospital. Eleven consecutive patients with POAG scheduled for treatment with a topical prostaglandin analog were included. The study protocol adhered to the tenets of the Declaration of Helsinki, was approved by the Institutional Review Board and Ethics Committee of Kyoto University Graduate School of Medicine, and was registered with the University Hospital Medical Information Network Clinical Trials Registry of Japan (ID, UMIN000015254; date of access and registration, September 25, 2014). Written informed consent was obtained from all patients.

The needed sample size for this study was calculated using the software G*Power 3.1 (http://www.gpower.hhu.de)^42^. We assumed the effect size as 0.4; α-error, 0.05; and power, 0.8, and obtained the needed sample size as 10. Taking some dropout cases into account, we decided to enroll 15 patients for this study.

The inclusion criteria were confirmed diagnosis of untreated POAG, best-corrected visual acuity (BCVA) ≥20/40, and age greater than 20 years old. Exclusion criteria were history of intraocular surgeries, usage of eye-drops, history of vitreoretinal diseases or non-glaucomatous optic neuropathy, and significant cataract. POAG was defined as open angle in gonioscopy and glaucomatous optic disc and visual field (VF) changes without any other ocular diseases or conditions that may elevate the IOP. Four glaucoma specialists (TA, HN, SM, and KS) evaluated eligibility of participants on the first visit. In cases where both eyes of a subject met the inclusion criteria, one of the eyes was randomly selected for the study.

All patients with POAG underwent comprehensive ophthalmic examinations including slit-lamp examination, IOP measurement with a Goldmann applanat ion tonometer, gonioscopy, uncorrected and BCVA with a Landolt chart, axial length measurements by partial coherence interferometry (IOLMaster; Carl Zeiss Meditec, Dublin, CA), standard automated perimetry (SAP) with Humphrey Visual Field Analyzer (24–2 Swedish Interactive Threshold Algorithm standard testing protocol; Carl Zeiss Meditec), spectral-domain OCT (Spectralis HRA + OCT; Heidelberg Engineering, Heidelberg, Germany), LSFG (LSFG-NAVI; Softcare Co., Ltd., Fukutsu, Japan), AOSLO, central corneal thickness with an ultrasonic pachymeter (SP-3000, Tomey, Tokyo, Japan), and systolic and diastolic blood pressure (sBP and dBP) at rest. The ocular perfusion pressure (OPP) was calculated as 2/3 [dBP+1/3 (sBP − dBP)] − IOP^30^.

To compare the measurements of three types of parameters in POAG eyes with those in normal eyes, eleven healthy eyes adjusted by age and axial length were used. Healthy eyes were defined on the basis of the following inclusion criteria: No evidence of retinal pathology or glaucoma, IOP < 21 mmHg, no chronic ocular or systemic corticosteroid use, an open angle on gonioscopy, and normal appearances of the optic disc and RNFL on photography and OCT examination. Subjects who had a history of systemic diseases such as diabetes mellitus or hypertension were excluded.

All POAG patients visited our clinic three times before initiating any treatment and underwent examinations of IOP, BP, LSFG, and AOSLO repeatedly at each visit. After baseline evaluations, they were introduced to topical tafluprost treatment and subsequently, underwent examinations of IOP, BP, AOSLO, OCT, and LSFG at 1 week, 4 weeks, and 12 weeks after starting topical tafluprost treatment. In order to minimize the effects of diurnal or seasonal variations, POAG patients were enrolled in the study in all seasons, and each examination was performed at almost the same time on each visit.

### Visual Field Assessment

We applied the criteria of Anderson and Patella for the detection of the glaucomatous VF results on SAP: Glaucoma hemifield test results outside the normal limits, pattern standard deviation (PSD) probability <5%, or a cluster of three or more adjacent non-edge points in typical glaucomatous locations that did not cross the horizontal meridian, all of which were depressed on the pattern deviation plot at P < 5%, and one of which was depressed at a level of P < 1% on at least two consecutive plots. The VF results were considered reliable at values of fixation loss ≤15%, false positive rate ≤15%, and false negative rate ≤15%.

### Adaptive Optics Scanning Laser Ophthalmoscopy Imaging and Parafoveal Blood Flow Velocity Measurements

The details of the AOSLO system developed by Canon (Canon, Inc., Tokyo, Japan) have been described previously^29, 43–45^. At the initial examination of each subject, AOSLO videos were obtained in the parafoveal area (approximately 0.25 to 0.50 mm from the foveal center) to entirely cover the innermost ring of the parafoveal capillary network (Fig. 1,B). Each high-resolution video was recorded for 2 seconds per scan area, with a field size of 1.4 × 2.8°. From the videos of multiple parafoveal regions, we chose a target capillary vessel that was in the innermost ring of the parafoveal capillary network, and free of bifurcations at the initial baseline examination for each patient. We also measured the same target vessel in subsequent follow-up examinations (Fig. 1,D). The AOSLO videos were recorded at a rate of 64 frames per second with a focus on the photoreceptor layer. The angle of each AOSLO image was converted to the actual distance on the retina on the basis of each patient’s axial length measurement using the AOSLO Retinal Image Analyzer (ARIA, Canon, Inc.) software produced by Canon.

The transparency of leukocytes to the AOSLO laser enables leukocytes to be identified as bright, moving objects, which represent light reflected from photoreceptors within the optical focus on the photoreceptor layer. The AOSLO laser does not pass through aggregated erythrocytes, which are depicted as black moving objects^44^. Blood components were identified via spatiotemporal (ST) images and pBFV was measured, using a previously described methodology^45^.

### Optic Disc Blood Flow Using Laser Speckle Flowgraphy

To evaluate relative optic nerve head (ONH) blood flow, we used LSFG-NAVI, which measured the pattern of speckle contrast produced by the interference of a laser scattered by blood cells moving in blood vessels. The principles of LSFG have been described in detail previously^23, 46^. We used LSFG Analyzer software (version 3.0.47.0; Softcare Co.) to calculate MBR in the optic disc area, which is a relative index of blood flow. The MBR_T_ was used for analyses because this measurement variable was reported to reflect capillary blood flow^26, 47^.

### Measurement of Retinal Vessel Diameter Using Optical Coherence Tomography

To measure inner diameters of retinal arteries and veins, a 12°-diameter-circle B-scan centered on the optic disc (circumpapillary scan) with Spectralis HRA+OCT was obtained by averaging 16 scans. Inner and outer walls of retinal vessels are observed as hyper-reflectivities in an OCT image, and we measured lumen diameters of retinal arteries and veins using an intrinsic software (Spectralis Acquisition and Viewing Modules, version 4.0; Heidelberg Engineering), as reported previously^22, 48^. We selected the largest artery and vein in the superotemporal and inferotemporal quadrants and measured the lumen diameters of these vessels in each quadrant. RVDs of an artery (RVD_A_) and a vein (RVD_V_) were defined as the average of lumen diameters of arteries and veins, respectively. This OCT system can identify previous scan location and guide the OCT laser to scan the same location using its follow-up function.

### Statistical Analysis

Statistical analyses were performed using the software EZR, which is based on R (http://www.r-project.org/)^49^. The decimal values of BCVA measured with the Landolt chart were converted to the logarithm of the minimal angle of resolution (logMAR) units for statistical analysis. Comparisons between the two groups were performed using the unpaired t-test. Differences in each parameter among all examination time points (baseline and 1, 4, and 12 weeks) were determined by repeated measures analysis of variance (ANOVA), and a post hoc test was performed to compare the measurement at baseline with each measurement after tafluprost initiation using the paired t-test. Significant differences in sampling distributions were determined using the chi-square test. All continuous values are presented as mean ± standard deviation. The level of statistical significance was set at *P* < 0.05, and in the post hoc test, statistical significance was determined after Bonferroni’s correction.

## Electronic supplementary material

Supplementary Video S1. Change of aggregated erythrocyte velocity before and after tafluprost induction

Dataset 2
